# Juxta-articular Myxoma of the Hand

**Published:** 2016-10-17

**Authors:** Shabaaz S. Sandhu, Joshua B. Elston, Michael A. Harrington, Wyatt G. Payne

**Affiliations:** ^a^Division of Plastic Surgery, Department of Surgery, University of South Florida Morsani College of Medicine, Tampa; ^b^C.W. Bill Young Bay Pines VA Medical Center, Bay Pines, Fla

**Keywords:** juxta-articular myxoma, hand mass, benign mass, local recurrence, ganglion cyst

## DESCRIPTION

**Figure 1:**
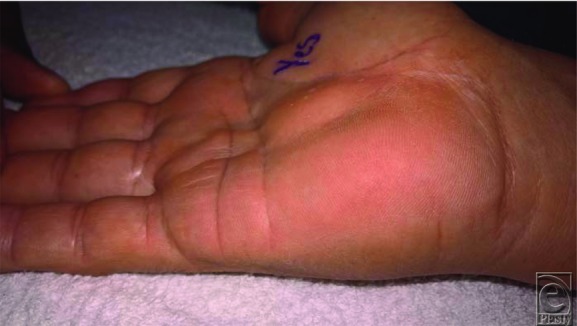
Preoperative view of the right hand demonstrating a compressible mass just distal to the prior carpal tunnel release incision. The mass was painless and did not produce tingling or numbness on Tinel's maneuver.

A 55 year-old right-hand-dominant man who developed asymptomatic, compressible swelling of the right hand 3 months after right open carpal tunnel release (Fig 1). Aspiration was unsuccessful. Magnetic resonance imaging demonstrated a 2 x 2-cm mass with an unclear diagnosis or origin. Excisional biopsy revealed juxta-articular myxoma (JAM).

## QUESTIONS

1. **What are the cause and pathogenesis of JAM?**
2. **How can JAM be differentiated from a ganglion cyst?**
3. **How does JAM differ from similarly fibroproliferative lesions on histology?**
4. **What are the treatment options and prognosis of JAM?**

## DISCUSSION

Myxomas are soft-tissue tumors of mesenchymal origin. They are benign but may demonstrate locally aggressive behavior with local recurrence after resection.^1^ The most common presentation of JAM is a swelling or mass (57%), which may be painful.^2^ JAM is a specific variant of myxoma associated with cyst formation often found in the vicinity of large joints, especially the knee (84%).^2^^,^^3^ Although the pathogenesis is not yet completely understood, JAM is most commonly associated with joint trauma and osteoarthritis. It is not currently thought to be associated with any specific syndromes and is more common in men in their third to fifth decades. It has been reported around the shoulder, elbow, foot, ankle, knee, and rarely in the hand/wrist.^2^^−^4 There is debate as to whether JAM is a neoplastic or reactive process, but this proliferative lesion is capable of extensive local tissue destruction.^1^^,^^2^^,^^4^

The presentation of JAM predisposes it to misdiagnosis, generally as a ganglion cyst or intramuscular myxoma; however, histological and clinical differences can be noted. Microscopically, ganglion cysts present a much less developed myxoid component. Ganglion cysts occur predominantly on the dorsal surface of the wrist or originate from the flexor carpi radialis tendon sheath of young women and are usually smaller in size than what is noted in JAM.^2^ Intramuscular myxomas are also more common in women and usually occur in the large muscles of the body (often the thigh and the shoulder). They display minimal cystic change and have a very low recurrence rate, even in cases of incomplete excision.^3^^,^^4^ As for any rapidly growing mass, the presentation of JAM might mimic that of malignant neoplasms, particularly sarcomas.^1^

Histologically, myxoid lesions are characterized by an abundance of extracellular mucinous material.^2^ There are typically a small number of spindle-shaped fibroblast-type cells found within this myxoid matrix, and vasculature is poorly developed.^3^ Macroscopically, the tumor is usually described as gelatinous, soft or friable, cystic, and pearly white to yellow-tan in color, with sizes usually ranging from 2 to 6 cm.^4^

JAM is usually managed via complete conservative surgical excision. Depending on the location and involvement of the lesion, additional procedures (such as meniscectomy) may be indicated to ensure comprehensive excision.^2^ The recurrence rate of JAM has been reported at 34% and usually occurs within 18 months.^4^ It has been suggested that incomplete resection of the JAM is responsible for this high rate.^3^ Recurrent lesions should be aggressively excised to minimize their reappearance, but the possibility of local recurrence must be anticipated and should be part of the preoperative counseling.^1^

**Figure 2:**
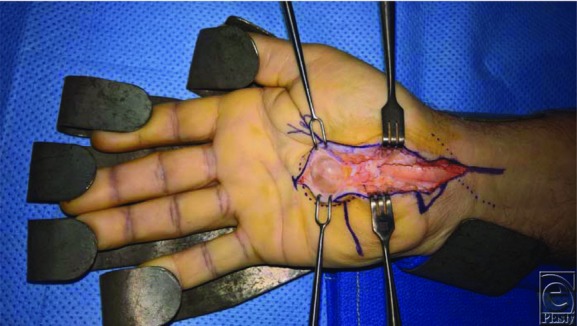
Intraoperative view after careful identification and protection of the right median nerve. The mass was clearly delineated and appeared to have a stalk originating outside of the carpal tunnel.

**Figure 3:**
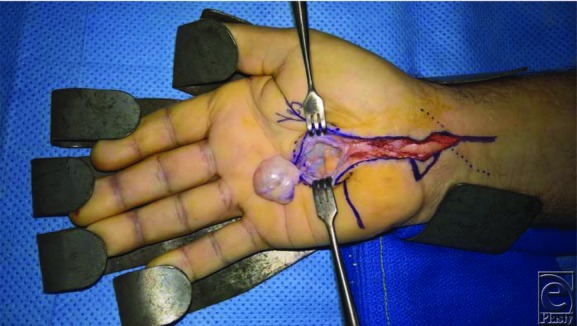
The mass reflected ex vivo with the stalk still attached to deeper structures.

**Figure 4:**
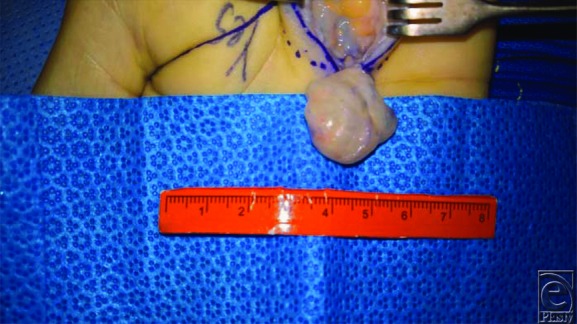
Closer view demonstrating well-circumscribed nature of the mass with the capsule intact for complete excision.

JAM is characterized by a benign soft-tissue tumor that is commonly found around the large joints of the body and rarely the hand/wrist. It is associated with joint trauma and osteoarthritis and usually presents as an enlarging, compressible mass, which may be painful. Imaging and histological analysis can be performed to differentiate JAM from other hand masses or malignant neoplasms. JAM is managed by complete surgical excision (Figs 2-4), but local recurrence is common and may cause further tissue destruction. These poorly circumscribed masses have been noted to enlarge rapidly, which may arouse suspicion of malignancy.^4^
